# Assessing the Effect of a Visual Navigational System on Route-Learning From an Ecological Perspective

**DOI:** 10.3389/fpsyg.2021.645677

**Published:** 2021-07-29

**Authors:** Harry Heft, Kelsey Schwimmer, Trenton Edmunds

**Affiliations:** ^1^Department of Psychology, Denison University, Granville, OH, United States; ^2^Educational Technology Services, Denison University, Granville, OH, United States

**Keywords:** route-learning, way-finding, navigation-systems, ecological psychology, tools

## Abstract

Route-learning, considered from an ecological approach to perception, is posited to involve the detection of information over time that specifies a path from one location to another. The study examines whether the use of a visual navigational system (e.g., GPS) may impede route-learning by drawing attention away from transitions along a path that serve as information for way-finding. Virtual reality (VR) technology used in conjunction with an extensive, detailed environmental simulation was employed to explore this possibility. One group of participants drove a simulated car in VR along a designated path while relying on visual GPS guidance. It was expected that use of the GPS display would draw attention away from temporally continuous path information. A second group initially drove the same route without GPS guidance. Both groups drove the path a second time without navigational assistance. Overall, the percentage of correct actions taken at intersections (transitions) during the second trial were significantly lower for the first group who initially drove the route with visual GPS guidance as compared to those who initially traveled the route without it. The results are consistent with the kind of trade-off that is commonplace when tools are used to mediate and assist skilled action.

## Introduction

This brief report describes an exploratory study of *route-learning* and subsequent *way-finding* designed from the theoretical perspective of ecological psychology. This perspective takes as the defining attribute of an organism its self-directed and selective engagement of the environment. There may be no more basic behavioral process for animate organisms than locomoting in some fashion from one location to another in their habitat. As such, the environment is engaged *over time*. The ecological approach proposes that there is *perceivable structure specific to a path of locomotion* that is revealed *over time* as the individual travels along a path. Such a formulation emphasizes the *dynamic character of perceptual experience* of the environment from the standpoint of an agent. Gibson (1966, 1979) developed this formulation in the context of his broader ecological approach to perception; and it was subsequently examined and explicated over a series of publications (Heft, 1983, 1996, 2012).

This conceptualization differs from a spatial cognition approach that has shaped most research about way-finding over the past half century. Early versions of that approach (e.g., Shemaykin, 1962; Siegal and White, 1975) posited that spatial knowledge that supports way-finding stems from the identification of paths and landmarks, with both serving as features used in *the construction of a mental representation* of the configuration of some region– a cognitive map. Subsequent navigation is said to draw upon the cognitive resources that the cognitive map makes available. This model has long influenced research and thinking in this research domain (e.g., Golledge, 1987; Evans, 1980), and continues to do so (e.g., Wolbers and Hegarty, 2010; Devlin, 2012; Bruns and Chamberlain, 2019).

Due to its focus on individual features, this paths-landmarks-cognitive map conceptualization necessarily neglects the temporal structure that accompanies traveling along paths. Historically, this feature-based approach derives from a theoretical lineage traceable at least to the 17th century that assumes visual experience to be constructed from discrete, static “images” (Boring, 1942; Pastore, 1971; Hatfield, 1990). At best, the temporal properties of perceptual experience are considered to be added *post hoc* to these “frozen” moments by supplemental processes. It is somewhat ironic that this template has been adopted so uncritically by environmental cognition researchers because what is widely considered to be a seminal contribution to this area is Lynch (1960) *The Image of the City* (1960) which highlights on its very first page the essential temporal dimension of moving through cities. In spite of that, and owing in part to Lynch’s own analysis, later researchers have focused exclusively on “imageable” features and properties of a cityscape, while disregarding the temporal character of environmental experience.

However, temporally based perceptual structures are commonplace in visual experience, as illustrated by evidence for the perception of any *inanimate and animate motion* (e.g., Johansson et al., 1980; Runeson and Frykholm, 1983) and for the perception of *optic flow* which accompanies *any* movement in relation to surfaces of the environment (Gibson, 1966; Lee, 1976). Is there a temporally based, visually perceivable structure accompanying locomotion relative to the environment that can be utilized in finding one’s way from one location to another?

Gibson (1979) proposed the following: Owing to the fact that environments tend to be cluttered with features (e.g., vegetation, landforms, built structures), and because there are changes in surface elevation, any given *vista* (i.e., a landscape region that can be seen from any observation point) does not visually extend indefinitely. A potential path across a vista goes out of sight at the edge of visual barriers created by features; as it extends over the crest of a hill; and with changes in direction of travel. As a result, as an individual travels along a path, a previously “out of sight” vista *gradually comes into view over time at the occluding edge of some feature*, such as a landform or a building or over the brow of a hill. This brief *duration* of experience when the “next” vista gradually comes into view is a *transition* in the path of travel. Unlike discrete features such as landmarks, a transition is a perceptual structure that emerges *in the course of* travel. It is revealed over time. A specific path of locomotion, then, from one location to another might be conceptualized as a particular sequence of transitions connecting successive vistas, that is, as an unfolding of a temporal event as vistas merge into adjacent vistas. Heft (1983, 1996) previously presented evidence in support of this conceptualization.

### Methodologies and Implementations

An obvious challenge in conducting research from this perspective is how to investigate route-learning and way-finding while preserving its temporal structure. Initial work in this vein was conducted *in situ* (Heft, 1979); but challenges such as unstable weather conditions prompted the use of filmed and later *videotaped* presentations of a path of travel (Heft, 1983, 1996, respectively). Also, these media formats could be edited to generate different presentation conditions.

However, it was apparent even then that these media have two notable limitations. First, when viewing films and videos, the individual is passively exposed to the path of travel. In recent decades researchers have attempted partially to address this limitation through the use of desktop computer (gaming) technologies that permit individuals to change viewing positions along paths within simulated environments (e.g., Peruch and Wilson, 2004; Gardony et al., 2013; van der Hamm et al., 2015). However, none of those studies have addressed their second limitation: paths of movement as experienced through these simulations are usually displayed within the two-dimensional boundaries of screen (e.g., a computer monitor). Perception of the path of travel in such cases is *spatially bounded* within a two-dimensional frame. As such, the individual remains a spectator looking onto a spatially circumscribed, if changing display, thereby undermining verisimilitude.

In the pilot study reported here, both of these limitations were partially surmounted through the use of virtual reality (VR) technology in conjunction with a rich and extended cityscape. The individual had control of his/her own movements through an extensive virtual environment which at all times surrounds the individual. The result is a compelling experience of moving around *within* the environment, rather than “looking on” at a two-dimensional framed display. VR technology has also been employed in a simulated cityscape by Bruns and Chamberlain (2019) and Weiner et al. (2020); but on less theoretically driven grounds where the temporal dimension of perceptual experience was not considered. Moreover, the virtual cityscape that was developed for the present research is far more extensive and detailed (see below) than that employed in earlier investigations.

### Tool Mediated Way-Finding

The use of VR in this study also permitted us to examine the effects of a *navigational tool* on route-learning. Tools, in general, mediate action. They facilitate the carrying out an action that would be otherwise difficult or prone to error in their absence. But the use of a tool commonly incurs a trade-off. When a tool is used, the individual may lose some of the prior acquired basic skills that the tool now mediates. In other cases, the availability of the tool short-circuits the need to develop those basic skills in the first place.

Such may be the case with the use of a navigational tool such as GPS. Anecdotal reports suggest that the burgeoning use over recent decades of navigational systems when driving, and even when biking and walking, would seem to indicate this tool can undermine route-learning processes. The experiment by Gardony et al. (2013) that employed auditory guidance cues broadly supports this possibility. They found that a navigational aid in the form of verbal instructions adversely affected spatial memory, as measured by tasks involving virtual pointing to locations not currently in view, map drawing task, and recall of landmarks. However, these measures assess *outcomes* only, while revealing little about how the verbal navigation cues affected on-going navigation over time. They did find that the auditory navigational enhanced path efficiency, that is, taking more direct paths between designated landmarks, but this measure was confounded by the fact that all participants were show an overhead view of the simulated environments before beginning the navigation task. Further, the simulated environments in the study were presented on a desktop monitor.

The use of VR technology in the present study created the possibility for an extended experience of environmental layout unimpeded by the constraints imposed through displays on computer monitors. It also made possible the use of a navigation aide that simulates the visual display of a GPS commonly used in driving. In combination, the implementation of VR along with a simulated GPS system allowed us to examine the potential effects of GPS on navigation from an ecological perspective.

Most significantly, nearly all studies of navigation and way-finding utilize outcome measures, asking participants, in effect, what they know about the environment or its features *after* having “traveled” through it. In this regard, these studies assess spatial memory, not performance. The present study – like others in our research program – assess the process of route-learning and way-finding as it is on-going.

### Summary and Rationale for This Investigation

From the perspective of ecological psychology, a route through the environment consists of a sequence of transitions connecting successive vistas. Transitions are perceived over time as one vista leads into the next. We employed a simulated visual GPS tool to test this claim by determining whether this tool, that is likely *to draw attention away from the temporal continuity of transitions*, would degrade subsequent way-finding. If this is the case, participants should make more errors when traveling a path that was previously learned with the aid of GPS.

## Method

### Participants

Twenty-one undergraduates at a Midwestern College in the United States participated in this study for course credit. Participants were between the ages of 18 – 22 and were recruited from an introductory psychology course. All but four participants were females. In research recruitment, prospective participants were told in advance that the experiment would involve the use of virtual reality (VR) and that those individuals who tend to experience adverse effects (e.g., dizziness, nausea) from simulation of self-movement on computer screens or in VR were discouraged from participating. IRB approval was obtained through standard departmental procedures.

### Materials

**FIGURE 1:**
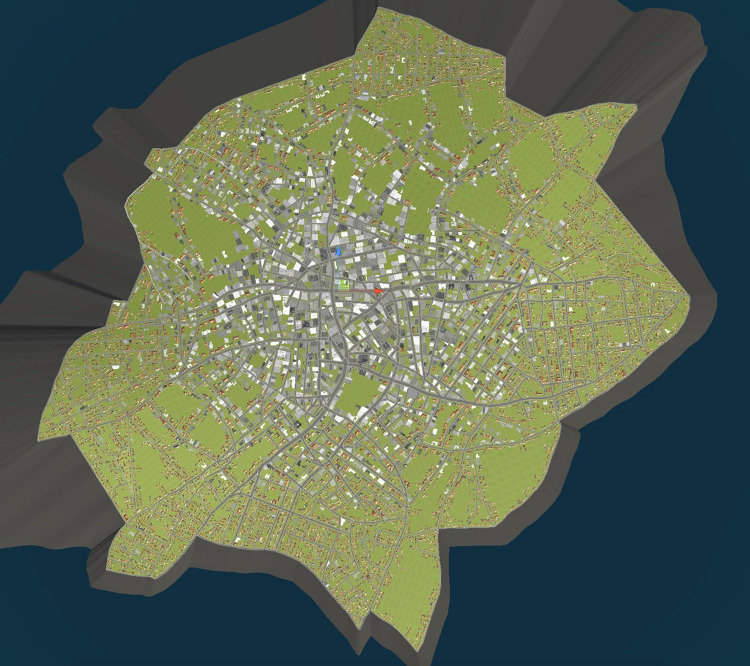
Overhead view of the city layout.

**FIGURE 2:**
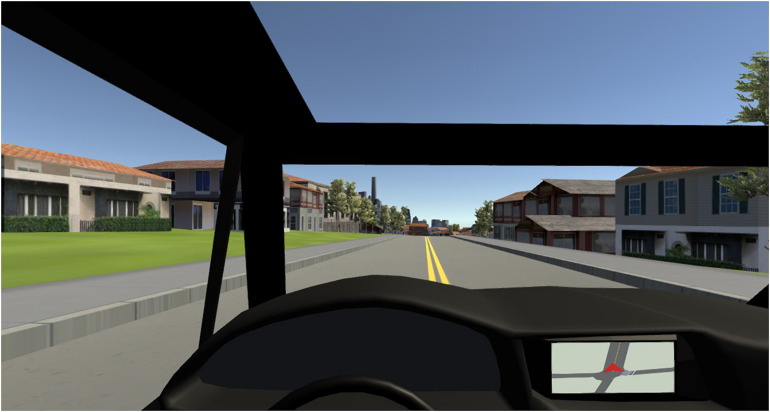
Driver’s eye view of console and road ahead.

A virtual city comparable in size to the metropolitan area of a Midwest USA city (area = 8,208 km^2^) was designed using Autodesk Maya for 3D models and editing, Autodesk City Engine for the framework of the city, and Unity software to create the car, navigation system, and lighting (see Figures 1, 2). The cityscape had a downtown core of high-rise office buildings, and a periphery of commercial areas and residential neighborhoods extending to the city limits. Building styles compatible to those different regions as well as some signage were present. Roadways of varying widths ran throughout, and trees were included. There were no other vehicles displayed on the roadways, nor were any pedestrians depicted. Slight topographical undulations throughout created minor hills and slopes for the roadways. The city was bounded by water, although it was not visible from the paths employed in this study. Whereas virtual environments created in other studies tend to have a high degree of regularity in layout with constant distances maintained between intersections and repetition of building facades (Bruns and Chamberlain, 2019; Weiner et al., 2020), variations in both of these respects modeling most everyday cityscapes were created for this study.

Participants had the capability of driving freely through the cityscape in VR by means of a Gameboy Controller for PC with which they could control direction and speed. They were positioned at the viewpoint of a driver behind a steering wheel seated in the front left-side seat (Figure 2). From that position, they could view ahead through the front windscreen, and views out of the side windows and rear were accessible with a turn of the head. There was also a console in the car to the right of the steering wheel on which a simulated GPS display could be presented. GPS displays typically present route information either from an aerial view or from eye-level (see Munzer et al., 2012 for a comparison of both presentation formats.) In the present study, an eye-level display was utilized.

The VR technology employed was an Oculus DK2, which employs a head-mounted display, using Windows 7 software and run on a Mac Pro tower. The GPS simulation that could be displayed on the console was created by yoking to the “camera” that moved with the car a second camera that was positioned outside of the field of view above the car and pointing down. The output of the second camera fed directly to the screen on the console.

### Procedure

**FIGURE 3:**
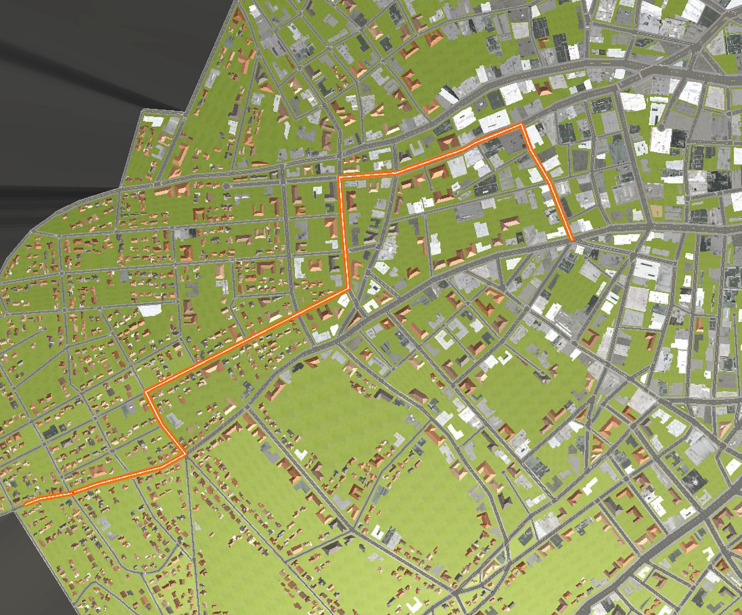
Route A (start is in lower left of the figure).

**FIGURE 4:**
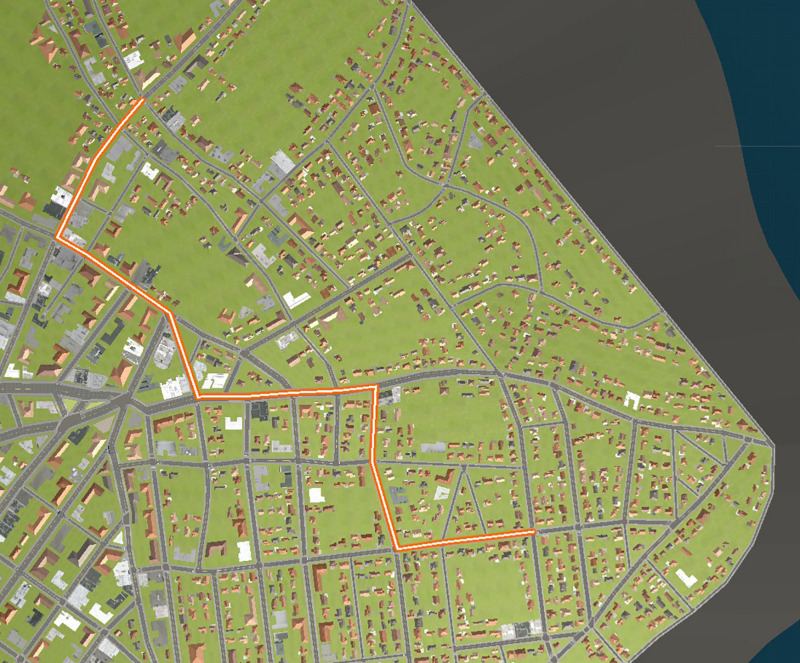
Route B (Start in lower right of the figure).

Two separate routes (A and B) were selected through the cityscape with 19 and 18 intersections, respectively (see Figures 3, 4). Participants were not shown these overhead displays. Participants were randomly assigned to one of the two routes for the duration of the experiment. Two routes were employed in order to be able to assess effects irrespective of a single route.

Participants were then instructed to place the VR goggles on their head, positioning them on their eyes and adjusting its fit as needed. Initially, they were given approximately 5 min to become acclimated to the VR display and to practice driving using the handheld controller. For this purpose, the experimenter positioned the car far from the experimental routes, and the participants were told that they could practice driving anywhere they wanted. The experimenter monitored their practice on an external computer screen. After 5 min of practice, all participants were able to control the car adequately.

The experiment consisted of two trials: a *training trial* to provide an opportunity to learn the designated route, followed by a 10-min break and then a test trial to assess way-finding performance. On both phases, the car was positioned at the start of a route (A or B) by the experimenter. Participants were randomly assigned to one of two conditions (GPS or No GPS) and to either route A or B. Because A and B included 19 and 18 intersections, respectively, and because performance on the two routes were combined in the analysis of performance within each experimental condition, assessment of route learning was evaluated based *the percentage of correct navigation choices relative to the total of possible choices per participant*. Participants drove the route at their own pace, and on average it took about 10 min to travel from start to finish on each trial.

In the “GPS experimental condition,” participants were instructed on the training trial to drive along the designated route by following a yellow double line that was superimposed on street along the path of travel until they reached a blue mailbox. The GPS tool provided visual guidance only. At the conclusion of the training trial, they removed the VR goggles for 10 min, completing two brief paper and pencil tasks. After that break, they replaced the VR goggles to find that the car had been repositioned by the experimenter at the start of the route they had previously traveled. The GPS display on the console was turned off. They were instructed to drive the car along the same route to the mail box as they had before, but in the absence of the GPS. The experimenter monitored their performance on a separate computer screen, and manually recorded their actions at each intersection. If the participant committed an incorrect navigational decision at an intersection, either by missing a turn or turning the wrong way, they were immediately corrected, directed back onto the route, and instructed to continue on.

In the “no GPS” condition, participants were asked to drive the route and to listen for the experimenter’s minimal verbal guidance as they did. They were unaware of the possibility of a GPS display on the console. The experimenter monitored their actions on an external computer screen, offering an occasional, intentionally brief comment such as “proceed ahead,” and just prior to a necessary turn, they were briefly instructed by the experimenter “to turn left/right” here. Care was taken to provide this limited auditory guidance at the last possible second before a turn. The intention was that their *hearing* the instruction just prior to the turn, and otherwise not knowing in advance when or if to alter direction, and in the absence of visual guidance from GPS, that they would otherwise not be distracted from the visual appearance of the route in the course of travel. After they reached the end of the route marked by the blue mailbox, the identical procedure employed in the GPS condition was followed. After a 10-min break during which two paper and pencil tasks were administered, they were instructed to replace the VR goggles and to drive the car once again along the same route that they followed on the previous trial, but on this trial no verbal cues were provided. The experimenter monitored their efforts on a separate computer screen and corrected any errors if necessary. Instances of errors committed at intersections were recorded during this test trial. Participants received a total score of correct navigational choices at each intersection.

The paper and pencil measures did not produce noteworthy results; and out of concern for length constraints required for this paper, their descriptions will be omitted here.

## Results

Ten individuals assigned to either route A or B participated in the GPS condition, and likewise eleven individuals assigned to A or B participated in the no GPS condition. Transitions in the path of locomotion, as defined above, occurred with the changes in direction at each intersection. The mean percentage of correct choices for participants in the GPS condition during the test trial was 71% (SD = 8.09), whereas the mean percentage correct for participants in the no GPS condition on the test trial was 84% (SD = 6.09). A univariate ANOVA revealed that participants who drove the route on the training trial without GPS guidance took correct actions at intersections a higher percentage of the time than those who initially drove the route using the GPS tool [*F*(1,20) = 17.50, *p* < 0.001, *d* = 1.81]. The relatively few males in the study mitigated meaningful comparisons of possible gender differences.

## Discussion

The results indicated that way-finding from the start to the end of the route on the test trial was comparatively less accurate for those participants who initially drove the route with reliance on the visual GPS tool in comparison to those who relied on minimal verbal directions from the experimenter. Based on the single performance index used – namely, correct actions at the intersections along the route – the data suggest at least one consequence that the use of GPS may have on route-learning: when visual attention is directed to the GPS display, individuals are not as attentive to the transitions over time – the hypothesized principal information for route-learning – than they would otherwise be. In short, as we see with the use of other tools, there may be a trade-off between, on the one hand, performance with the aid of a tool and, on the other, what the users come to learn in its absence. More generally, the degradation of performance as measured by actions at the transitions between vistas is consistent with expectations based on with the ecological conceptualization of way-finding.

Even though performance on the test trial differed across experimental conditions, it was somewhat better in the GPS condition than the experimenters anticipated at the outset. We expect that performance differences between groups such as these would widen if the path of travel were lengthened and the number of choice points increased.

This preliminary study warrants additional and more extensive empirical verification, especially in view of the relatively small samples tested. One potential useful direction to take would be to add virtual locomotion to the simulation (Templeman et al., 1999) in order to more fully simulate the experience of everyday perception-action in the course of way-finding.

In spite of obvious limitations, e.g., small sample size, this investigation should be viewed as an extension of a research program that has operated intermittently over several decades. The common thread is an ecological approach to perceiving that emphasizes the agency of perceivers and the temporal nature of perceptual experience (Heft, 2012). We hope that as modest as it is, this preliminary study can be a model for further research on navigation and spatial cognition from an ecological perspective.

## Data Availability Statement

The raw data supporting the conclusions of this article will be made available by the authors, without undue reservation.

## Ethics Statement

The studies involving human participants were reviewed and approved by the Denison University Psychology Department. The patients/participants provided their written informed consent to participate in this study.

## Author Contributions

HH designed the experiment, prepared the research report and approved the final version. KS collected and analyzed the data. TE provided the essential technical contributions.

## Conflict of Interest

The authors declare that the research was conducted in the absence of any commercial or financial relationships that could be construed as a potential conflict of interest.

## Publisher’s Note

All claims expressed in this article are solely those of the authors and do not necessarily represent those of their affiliated organizations, or those of the publisher, the editors and the reviewers. Any product that may be evaluated in this article, or claim that may be made by its manufacturer, is not guaranteed or endorsed by the publisher.
